# D2D Mobile Relaying Meets NOMA—Part I: A Biform Game Analysis

**DOI:** 10.3390/s21030702

**Published:** 2021-01-20

**Authors:** Safaa Driouech, Essaid Sabir, Mounir Ghogho, El-Mehdi Amhoud

**Affiliations:** 1 NEST Research Group, LRI Lab., ENSEM, Hassan II University of Casablanca, Casablanca 20000, Morocco; safaa.driouech@sorbonne-universite.fr; 2 Laoratoire de Reacherche en Informatique, Sorbonne Université, CNRS, LIP6, F-75005 Paris, France; 3 Department of Computer Science, University of Quebec at Montreal, Montreal, QC H2L 2C4, Canada; 4 TICLab, International University of Rabat, Rabat 11100, Morocco; mounir.ghogho@uir.ac.ma; 5 School of Computer Science, Mohammed VI Polytechnic University, Ben Guerir 43150, Morocco; elmehdi.amhoud@um6p.ma

**Keywords:** D2D-relaying, 5G/B5G/6G, biform game, self-organized devices, Nash equilibrium, distributed reinforcement learning, NOMA/OMA

## Abstract

Structureless communications such as Device-to-Device (D2D) relaying are undeniably of paramount importance to improving the performance of today’s mobile networks. Such a communication paradigm requires implementing a certain level of intelligence at device level, allowing to interact with the environment and select proper decisions. However, decentralizing decision making sometimes may induce some paradoxical outcomes resulting, therefore, in a performance drop, which sustains the design of self-organizing, yet efficient systems. Here, each device decides either to directly connect to the eNodeB or get access via another device through a D2D link. Given the set of active devices and the channel model, we derive the outage probability for both cellular link and D2D link, and compute the system throughput. We capture the device behavior using a biform game perspective. In the first part of this article, we analyze the pure and mixed Nash equilibria of the induced game where each device seeks to maximize its own throughput. Our framework allows us to analyse and predict the system’s performance. The second part of this article is devoted to implement two Reinforcement Learning (RL) algorithms enabling devices to self-organize themselves and learn their equilibrium pure/mixed strategies, in a fully distributed fashion. Simulation results show that offloading the network by means of D2D-relaying improves per device throughput. Moreover, detailed analysis on how the network parameters affect the global performance is provided.

## 1. Introduction

### 1.1. Motivations & New Trends

The last twenty years have known a noteworthy growth in the demand for more network capacity. This was mainly caused by the unprecedented Internet built-out and the huge traffic generated by a massive number of devices. To cope with this neverseen demand, substantial research effort is being conducted to enhance the performance of next generation mobile networks. Current fifth Generation (5G) of wireless networks addresses a wider range of applications and many innovative use-cases [1]. It is expected that a single 5G tower will serve up to 1 Million device per km${}^{2}$, which generates massive data traffic in cellular networks. The sixth Generation (6G) of wireless networks is foreseen to support novel data-hungry applications, a plethora of autonomous services and new communication scenarios around 2030. These technologies encompass holographic videos, flying networks and vehicles, teleoperated driving, telemedicine, haptics, human bond communications, brain-computer interfaces, connected autonomous systems, high-definition video streaming and the tactile Internet, to name a few. Thus, the volume of wireless data traffic and the number of connected objects are expected to increase hundred-folds in a given cubic meter [2]. 6G will connect millions of users and billions of machines everywhere through the emergence of the Internet of Everything (IoE) ecosystem. Thus, many strict requirements need to be met such as low energy consumption, long battery life, high intelligence, extremely larger bandwidth than 5G (The THz band is defined from 0.1 THz to 10 THz), high reliability, low latency, and high data rates, etc. [3,4,5,6].

Device-to-Device (D2D) relaying has been proposed as an efficient solution to lower energy consumption and extend the battery life of the mobile device, while expanding the network coverage and improving local performance in a rapid and cost-effective way. This is met by offloading the traffic to devices exhibiting better channel conditions. D2D communication allows devices to communicate directly between each other instead of going through the Base Station (BS) [7,8]. Unfortunately, the infrastructureless nature of D2D communications raises challenges on how to efficiently integrate D2D communication within the current cellular ecosystem. Yet, a D2D communication requires lower transmission power for mobile devices, and improves network performance both under inband and outband schemes. Nonetheless, a large number of D2D users may induce higher uncontrollable interference and might lead to capacity failure at relay level. This is why it is crucial to strategically set the devices that need to use D2D links and those that need to be relays within a cell.

Under legacy networks, the BS needs to have complete information about the network and the active devices. Then, it computes the optimal parameters and the best Radio Access Network (RAN) association. Next, it remotely configures devices through a heavy signaling. However unfortunately, such a centralized system is known to suffer from a heavy overhead and complex signaling mechanisms, under massive environments, which greedily misuse the network resources. Consequently, a fully distributed system is recommended for dense/ultra-dense networks, as it offloads the network and minimizes the dependency on its connectivity.

Nowadays, we live in a hyper connected world where the performance of each device is mutually affected by decisions taken by the other devices. Hence, to opt for the best strategies under partial information, a decentralized scheme is the natural solution. Moreover, decentralizing decision-making exhibits promising scalability features and can efficiently avoid server break-downs due to unsupported number of requests. To ensure a distributed system with ubiquitous intelligence, self-organized devices are by design the master-pieces. It is an autonomous system designed to enable the devices automated resource management, diminishes BSs tasks, reduces human intervention, and optimizes available resources, etc. The general aim of this work is to offload the BS and avoid the system breakout, via self-organized devices implementing some Artificial Intelligence (AI) techniques and decentralized Machine Learning (ML) algorithms. The updating pattern at the device level only requires local actions and observed/measured payoffs perceived. Such an adaptive algorithm is very important in dynamic/stochastic environments where many parameters are unavailable, unobservable or simply unknown.

Today’s 5G networks underuse artificial intelligence and machine learning, which results in poor self-organizing capabilities. In contrast, it is foreseen that AI/ML will be the signature for 6G for smarter and more powerful networks, as it will penetrate network, service, content and user equipment’s. Everything will be very intelligent, giving rise to the concept of IoE, with an enormous amount of data and information. AI-empowered 6G is believed to be able to provide a series of all new features, e.g., network decentralization, self-organization, context-awareness, self-configuration and self-healing properties. It will also enable reliable device-to-device (D2D) communications in a fully intelligent way [3,5]. Unfortunately, self-organized devices could make sub-optimal decisions, lead to unwanted and unexpected/paradoxical results. Thus, it is of paramount importance to make sure the devices are reasoning properly and converge to efficient operation points with satisfactory performance, the network should be carefully designed. This study could be done either through test beds or real implementations and/or simulations which are costly and time consuming. In this work, we analyze the network by means of game theory. In other words, a network designer (e.g., the network builder or the operator) will analyze the performance of the network and predict its operation points using game theory before rolling it out. The benefits of game theory rely on providing strong tools and theoretic framework to analyze the agents/devices interaction. This allows us to accurately predict the system performance. Game theory is useful in predicting the network performance while considering self-organized devices. Henceforth, the network designer can build efficient mechanisms granting the whole system to run properly under almost a zero-touch paradigm.

### 1.2. Our Contributions

To allow User Equipment (UE) to communicate and connect to the BS, a multiple access technology is utilized. Multiple access techniques can broadly be categorized into two different approaches, namely, Orthogonal Multiple Access (OMA) and Non-Orthogonal Multiple Access (NOMA). On one hand, OMA allows UEs to use orthogonal signals to eliminate interference, such as Orthogonal Frequency-Division Multiple Access (OFDMA) used in 4G mobile networks. On the other hand, NOMA is envisioned to be used as a candidate radio access technology for beyond 5G and 6G cellular systems. It allows allocating one frequency channel to multiple users at the same time within the same cell either in the power domain or the code domain. Moreover, NOMA offers a number of advantages, including improved spectral efficiency, enhanced resource allocation, higher cell-edge throughput, and lower latency (no scheduling request from users to base station is required) [9,10,11].

In a nutshell, we use game theory in the first part of this article to analyze and solve the conflict of interest raised between self-organized devices. The individual average throughput is considered as the payoff function. More precisely, we build a biform game, for which we analyze the pure/mixed Nash equilibria. The second part of our work [12] presents two distributed reinforcement learning algorithms to be implemented at the device level in order to reach equilibrium strategies. Our mechanism is robust as it is based on Nash equilibrium concept, and reduces the risk of bad decisions, allowing thereby to benefit from appreciated self-organizing and self-configuring features.

The main contributions of this work are fivefold:

**Part I’s** contributions are related to performance analysis of a self-organizing D2D relaying scheme:

1. We consider a hybrid two-tier scheme where cellular links use NOMA, whilst D2D links use OMA. This scheme is suitable for both inband and outband D2D schemes;
2. We fully characterize the Rayleigh channel model and derive closed forms for the outage probability of both OMA and NOMA links, and then compute the average throughput perceived by each device in the network;
3. To the best of our knowledge, this work is the first to implement a biform game to capture the devices’ behaviors while deciding which Radio Access Network (RAN) to connect. In order to evaluate the outcome of the game, detailed analysis of pure and mixed Nash equilibria are provided for 3-person game and generalized to n-person game;

**Part II’s** contributions are related to implementing a self-organized mode selection using RL:

4. We propose to empower devices with a self-organize capability allowing to reach pure Nash equilibria (Linear-Reward Inaction) and mixed Nash equilibria (Boltzmann-Gibbs dynamics), in a fully distributed manner;
5. We perform extensive simulations to analyze the effect of different parameters on the learning schemes. Insights on accuracy and convergence are also provided.

The rest of this article is organized as follows: A comprehensive literature review is presented in Section 2. The problem is formulated in Section 3. We provide a full equilibrium analysis for the 3-person game in Section 4. The general case of n-player game is discussed in Section 5. Numerical investigations are presented in Section 6. Finally, we draw some concluding remarks and list future works in Section 7. Part II [12] of this research, presents the proposed decentralized reinforcement learning algorithms and access their dynamics and performance.

## 2. Related Work

D2D communications are widely used to relay information and improve local/overall performance by offloading traffic to other devices in the network, extending system coverage, mitigating wireless fading through improving the capture effect and exploiting spatial diversity. Also, reducing transmit power allows us to lower the impact of cross-interference, which helps to improve the network performance, enhance the QoS (improved throughput, reduced latency, and increased reliability) [13,14,15]. In latter researches, and in most of D2D published papers, a great attention is given to the performance enhancement of other technologies by introducing D2D communication (e.g., IoT [13] and Massive MIMO Systems [15]). In our article, we discuss the importance of strategically selecting the best RAN (i.e., either cellular or D2D) according to the network status, in a way to improve the devices experienced QoS. Game theory is a set of applied mathematical tools aiming to understand and solve decision-making problems, such as competing and independent actors during conflicts. It has been extensively used in wireless networks [16,17], and more specifically in solving cooperation and competition problems between devices over limited resources [18,19,20,21,22,23,24,25,26].

In the last few years, a tremendous research effort has been conducted in order to adapt and adopt self-organized networks. Self-organizing resource management approaches have attracted attention because of their low complexity, scalability and their important role in reducing information exchange [27]. It has been investigated for various networks from different perspectives including learning mechanisms, heuristic and game-theoretic approaches [28,29,30]. The authors in [28] propose a distributed utility-based SINR adaptation at small-cells that diminishes the cross-tier interference. The authors in [29] carry out a comparison among two decentralized heuristic algorithms, with no involvement of any centralized entity, for joint power assignment and resource allocation in small-cells. In [30], the authors present an energy-efficient self-organized cross-layer optimization scheme where each D2D transmitter strategically selects the resource blocks and the power levels for improving its energy efficiency while maintaining a certain QoS requirement of other tiers. However, the autonomy and self-organization of autonomous collaborative networks of devices make them especially vulnerable to attacks. Thus, such a network needs a dependable mechanism to detect and identify attackers and enable appropriate reactions. That is why the authors in [31] propose a scalable adversary detection for autonomous networks, a scheme to efficiently identify malicious devices within large networks of collaborating entities. It is designed to run in truly autonomous environments, i.e., without a central trusted entity. Unlike related works on D2D mode selection appended in Table 1, where authors focus on optimizing the network performance, we aim to model and understand the interplay between D2D, NOMA and OMA. Then, we use biform game theory to predict, and decentralized machine scheme to learn what options each device should pursue to earn the “best” long-term average profit.

## 3. System Model

**Figure 1:**
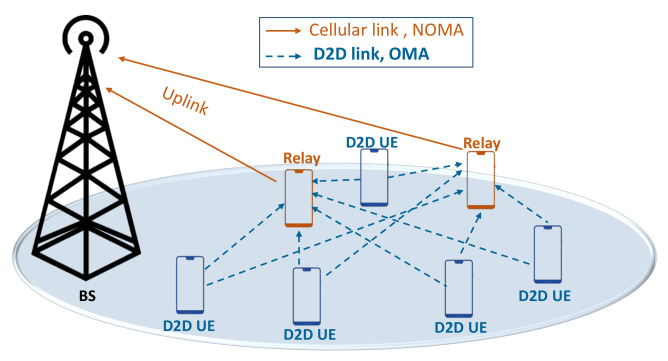
Cellular offloading using D2D cooperative relaying.

Consider the uplink case of a single 4G/5G/6G cell, where a finite number of devices N={1,2,….,n}, are randomly distributed around the serving BS. The devices communicate using NOMA in cellular links combined with conventional OMA for D2D links as shown in Figure 1. We use a separate band for D2D users (i.e., D2D overlay mode). We use OMA for D2D links to (1) study a hybrid access system; and (2) eliminate interference effect between cellular and D2D UEs. Here, we use stochastic geometry to estimate the performance of D2D users. Each device i∈N transmits its data to the BS using power $P_{i}$ from a distance $d_{i}$ while experiencing a channel gain $h_{i}$. For better readability, the main notations and symbols used in this article are listed in Table 2.

For the sake of simplicity and without loss of generality, device numbered 1 is the closest device to the BS, with distance $d_{1}$. It transmits with the lowest power $P_{1}$ and experiences the strongest channel $h_{1}$. Whilst device *n* is the farthest with distance $d_{n}$ from the BS, uses the highest transmission power $P_{n}$ and experiences the poorest channel $h_{n}$. Namely, we have $|h_{1}{|}^{2}\geq |h_{2}{|}^{2}\geq \cdots \geq |h_{n-1}{|}^{2}\geq {\left|h_{n}\right|}^{2}$. Let w(t) be the received noise at the BS and assume each device *i* transmits its individual signal $s_{i}\left(t\right)$. Then, the aggregate received signal at the BS writes:

(1) $$S\left(t\right)=\sum_{i=1}^{n}\sqrt{P_{i}}h_{i}s_{i}\left(t\right)+w\left(t\right),$$

The BS decodes the signals by applying the Successive Interference Cancellation (SIC) technique [41,42]. The received signal power corresponding to the strongest channel user is likely the strongest at the BS and is therefore the first to be decoded at the BS and experiences interference from all the remaining weaker channels’ users in the cluster. So, the transmission of device 1 experiences interference from users with weaker channels in the cluster, whereas the transmission of device n experiences zero interference. In contrast to downlink NOMA, each user in uplink NOMA can independently utilize its battery power up to the maximum since the channel gains of all the users are sufficiently distinct [43].

### 3.1. Channel Model

Within this article, the radio signal experiences attenuation due to the path-loss with exponent α and a Rayleigh fading. We denote by $\gamma_{i}$ the instantaneous Signal-to-Interference-and-Noise-Ratio (SINR) of device *i*, which is given by:

(2) $$\gamma_{i}=\frac{P_{i}{\left|h_{i}\right|}^{2}d_{i}^{-\alpha }}{\sum_{j=i+1}^{n}P_{j}{\left|h_{j}\right|}^{2}d_{j}^{-\alpha }+\sigma_{N}^{2}},$$

It is worth nothing that the SINR of the weakest device *n* experiences no interference according to NOMA operation, i.e., $\gamma_{n}=\frac{P_{n}{\left|h_{n}\right|}^{2}d_{n}^{-\alpha }}{\sigma_{N}^{2}}$. $\sigma_{N}^{2}$ denotes the variance of the thermal additive white Gaussian noise. Through this article, each device aims at guaranteeing an instantaneous SINR above a certain threshold $\gamma_{i,th}$ to have successful communication. The outage probability denotes the probability that the SINR is less or equal than a given SINR threshold ($\gamma_{i,th}$). It is calculated as follows:

(3) $$\begin{array}{ccc} P_{i}^{out}\left(\gamma_{i}\right) & = & Pr\left(\gamma_{i}\leq \gamma_{i,th}\right)=Pr\left(\frac{P_{i}{\left|h_{i}\right|}^{2}d_{i}^{-\alpha }}{\sigma_{N}^{2}+\sum_{j=i+1}^{n}P_{j}{\left|h_{j}\right|}^{2}d_{j}^{-\alpha }}\leq \gamma_{i,th}\right)\\ & = & Pr\left(|h_{i}{|}^{2}\leq \frac{\gamma_{i,th}\sigma_{N}^{2}}{P_{i}d_{i}^{-\alpha }}+\frac{\gamma_{i,th}}{P_{i}d_{i}^{-\alpha }}\sum_{j=i+1}^{n}P_{j}{\left|h_{j}\right|}^{2}d_{j}^{-\alpha }\right)\\ & = & \int_{0}^{+\infty }f_{|h_{n-1}{|}^{2}}\left(x_{n-1}\right)\int_{0}^{+\infty }f_{|h_{n-2}{|}^{2}}\left(x_{n-2}\right)\cdots \int_{0}^{+\infty }f_{|h_{1}{|}^{2}}\left(x_{1}\right)\int_{0}^{A}f_{|h_{i}{|}^{2}}\left(x_{i}\right)\;dx_{1}dx_{2}\cdots dx_{n-1}. \end{array}$$

with $A=\frac{\gamma_{i,th}\sigma_{N}^{2}}{P_{i}d_{i}^{-\alpha }}+\frac{\gamma_{i,th}}{P_{i}d_{i}^{-\alpha }}\sum_{j=i+1}^{n}P_{j}{\left|h_{j}\right|}^{2}d_{j}^{-\alpha }$. Assuming that all channels undergo Rayleigh fading, the channel power gain ${\left|h\right|}^{2}$ is an exponential random variable with PDF $f_{{\left|h\right|}^{2}}\left(x,\lambda \right)=\lambda e^{-\lambda x}$, where $\frac{1}{\lambda }$≥ 0 is the mean and scale parameter of the distribution, often taken equal to 1. Therefore, the outage probability can be expressed as:

(4) $$P_{i}^{out}\left(\gamma_{i}\right)=1-\frac{\prod_{j=i+1}^{n}\lambda_{j}.e^{-\frac{\gamma_{i,th}\sigma_{N}^{2}\lambda_{i}}{P_{i}d_{i}^{-\alpha }}}}{\prod_{j=i+1}^{n}\left(\lambda_{j}+\frac{\gamma_{i,th}P_{j}d_{j}^{-\alpha }}{P_{i}d_{i}^{-\alpha }}\lambda_{i}\right)}$$

### 3.2. Average Throughput

In general, device *i* transmits data with a rate $R_{i}$ in every channel use (i.e., in every packet or frame transmission), in a condition that $R_{i}$ must not exceed its channel capacity, i.e., $R_{i}\leq \log\left(1+\gamma_{i}\right)$. We define the throughput of the transmission as the rate of successful data bits that are transmitted to the destination over a communication channel. As the channel is variable, random and unknown, the throughput of device *i* is a function of the outage probability $P_{i}^{out}\left(\gamma_{i}\right)$ that depends on the average of the channel gain, expressed as follows:

(5) $$\Theta_{i}\left(\gamma_{i}\right)=\frac{M}{L}R_{i}\left(1-P_{i}^{out}\left(\gamma_{i}\right)\right)=\rho_{i}\left(1-P_{i}^{out}\left(\gamma_{i}\right)\right),$$

with $\rho_{i}=\frac{M}{L}R_{i}$. *M* is the data length. *L* denotes the total number of bits in a frame with *L* = *M* + *H* data bits, and *H* is the length of the header.

### 3.3. Biform Game Analysis

The main goal of game theory is to study the strategic relations between rational players that strive to maximize their payoffs in the game and where the actions and choices of all the players affect the outcome of each player. In this work, the devices inside the cell decide either to communicate through the cellular link or to switch to D2D communication. Each device aims at making a decision that allows it to maximize its throughput. However, since each device decision influences the throughput of the other devices, we are concerned here about finding an equilibrium point and a prediction of what options players may take to earn the best profit. For this purpose, we use biform game theory. Biform game is a two-stage game that combines a competitive and cooperative game in one formal model. In the first stage, decisive players choose their strategies in a non-cooperative way to maximize their expected payoffs. Each profile of strategic choices at the first stage leads to the second stage, which is a cooperative game, where the actual payoff is realized. This gives the competitive environment created by the choices of the players in the first stage [44,45].

Let G = {N,${\left\{A_{i}\right\}}_{i\in N}$,${\left\{U_{i}\right\}}_{i\in N}$} be a biform game. N is the set of players of G. $A_{i}$ is the set of actions of each player *i*, either to be a relay $a_{i}=0$ or to communicate through D2D $a_{i}=1$. $U_{i}$ is the payoff of each device *i* that represents its throughput. There are two cases of modeling the problem:

- The first case is to consider the game from the perspective of one of the players, and define what is the action that each player needs to take to maximize its throughput depending on the network parameters and on the other players’ probabilities of relaying.
- The second case is to consider the problem from an equilibrium perspective. In fact, we need to seek for the equilibrium probability vector where no player has incentive to deviate unilaterally. In this case also, each player could attain its maximum utility function at the equilibrium, depending on its own strategy and the strategy of other players.

## 4. Equilibrium Analysis for the Three-Player Game

**Figure 2:**
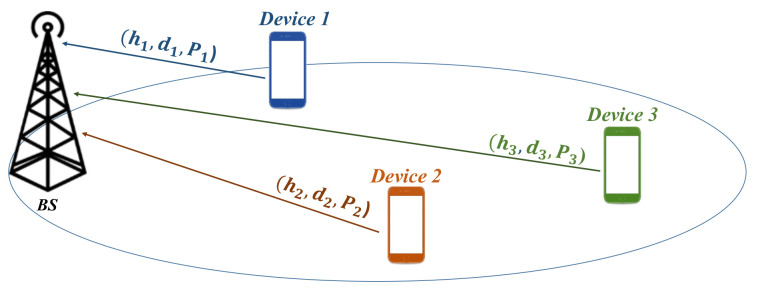
Network model for three−device case.

Consider a three devices power-domain NOMA operation in a single cell network. Each device is communicating through uplink as shown in Figure 2.

Each device i={1,2,3}, is transmitting its data to the BS with a power $P_{i}$, from a distance $d_{i}$ and with $h_{i}$ as the channel coefficient between device *i* and the BS.

### 4.1. Channel Model

Let us consider device 1 as the closest to the BS with the lowest transmit power $P_{1}$, the smallest distance $d_{1}$ and best channel condition $h_{1}$. Device 3 has the farthest distance $d_{3}$ from the BS with the highest transmit power $P_{3}$, and the weakest channel gain $h_{3}$.

Device 1 is considered as the strongest device experiencing the strongest channel, while device 3 is the weakest. According to the conventional uplink NOMA operation, the BS successively decodes and cancels the signal of device 1 that experiences interference from the two other devices, then device 2 which is affected only by interference of device 3 and finally decodes the signal of device 3 that experiences zero interference. Each device’s SINR is then expressed as:

(6) $$\gamma_{1}=\frac{P_{1}{\left|h_{1}\right|}^{2}d_{1}^{-\alpha }}{P_{2}|h_{2}{|}^{2}d_{2}^{-\alpha }+P_{3}{\left|h_{3}\right|}^{2}d_{3}^{-\alpha }+\sigma_{N}^{2}},\;\gamma_{2}=\frac{P_{2}{\left|h_{2}\right|}^{2}d_{2}^{-\alpha }}{P_{3}{\left|h_{3}\right|}^{2}d_{3}^{-\alpha }+\sigma_{N}^{2}},\;\gamma_{3}=\frac{P_{3}{\left|h_{3}\right|}^{2}d_{3}^{-\alpha }}{\sigma_{N}^{2}}$$

The outage probability of each device *i*, is given by:

(7) $$\begin{array}{cc} \; & P_{1}^{out,c}\left(\gamma_{1}\right)=1-\frac{\lambda_{2}\lambda_{3}\;e^{-\frac{\gamma_{1,th}\sigma_{N}^{2}\lambda_{1}}{P_{1}d_{1}^{-\alpha }}}}{\left(\lambda_{2}+\frac{\gamma_{1,th}P_{2}d_{2}^{-\alpha }}{P_{1}d_{1}^{-\alpha }}\lambda_{1}\right)\left(\lambda_{3}+\frac{\gamma_{1,th}P_{3}d_{3}^{-\alpha }}{P_{1}d_{1}^{-\alpha }}\lambda_{1}\right)},\\ \; & P_{2}^{out,c}\left(\gamma_{2}\right)=1-\frac{\lambda_{3}\;e^{-\frac{\gamma_{2,th}\sigma_{N}^{2}\lambda_{2}}{P_{2}d_{2}^{-\alpha }}}}{\left(\lambda_{3}+\frac{\gamma_{2,th}P_{3}d_{3}^{-\alpha }}{P_{2}d_{2}^{-\alpha }}\lambda_{2}\right)},\;P_{3}^{out,c}\left(\gamma_{3}\right)=1-e^{-\frac{\gamma_{3,th}\sigma_{N}^{2}\lambda_{3}}{P_{3}d_{3}^{-\alpha }}}. \end{array}$$

### 4.2. Throughput

At each time slot, each device can choose to communicate through cellular and serves as a relay or, communicate through D2D. D2D links use OMA as a multiplexing access method. Also, the D2D transmitters operate in an overlaying mode, where D2D and cellular devices are allocated distinct frequency resources which enables to suppress interference between cellular and D2D devices. Depending on the devices choices, each device *i* earns a throughput and experiences an outage probability as follows:

- If all the devices communicate through cellular mode, then the throughput of each device is:
  (8) $$Thp_{i}^{c}=\Theta_{i}^{c}\left(\gamma_{i}\right)=\frac{M}{L}R\left(1-P_{i}^{out,c}\left(\gamma_{i}\right)\right)=\rho \left(1-P_{i}^{out,c}\left(\gamma_{i}\right)\right).$$
  We suppose that the BS allocates the same transmit rate R to all devices. For each device *i*, $P_{i}^{out,c}\left(\gamma_{i}\right)$ is defined in Equation (7).
- If device *i* decides to be a relay while devices *j* and *k* transmit through D2D, i,j,k∈{1,2,3}, then:
  (9) $$\left\{\begin{array}{c} Thp_{i}^{c,d}=x_{i}\Theta_{i}^{c,d}\left(\gamma_{i}\right)=x_{i}\rho \left(1-P_{i}^{out,cd}\left(\gamma_{i}\right)\right),\\ P_{i}^{out,cd}=1-e^{-\frac{\gamma_{th}\sigma_{N}^{2}\lambda_{i}}{P_{i}d_{i}^{-\alpha }}} \end{array}\right.\left\{\begin{array}{c} Thp_{j}^{d}=\frac{\left(1-x_{i}\right)}{2}\rho \left(1-P_{i}^{out,cd}\left(\gamma_{i}\right)\right)\left(1-P_{j}^{out,d}\left(\gamma_{j}\right)\right),\\ P_{j}^{out,d}=1-\frac{\lambda_{k}\;e^{-\frac{\gamma_{th}\sigma_{N}^{2}\lambda_{j}}{P_{j,d}d_{j,d}^{-\alpha }}}}{\lambda_{k}+\frac{\gamma_{th}f_{j,k}P_{k,d}d_{k,d}^{-\alpha }}{P_{j,d}d_{j,d}^{-\alpha_{d}}}\lambda_{j}} \end{array}\right.$$

If there is at least one device in the D2D group, then the relay device allocates a fraction of its throughput $x_{i}$ to that group. $x_{i}$ allows also to define the mode selection of device *i*. For instance, $x_{i}=1$ means device *i* fully opts for cellular mode. Meanwhile $x_{i}=0$ means device *i* chooses to communicate through D2D link. When $x_{i}\in ]0,\;1[$ the device *i* plays the role of a relay. Here, we assume that the fraction given from the relay will be equally divided between the devices in D2D mode. $P_{j,d}$ and $d_{j,d}$ are the transmit power and the distance of the D2D device *j*, respectively. The power transmission in cellular communication is much higher than the D2D transmit power because of the short distances between D2D devices in comparison with the distances between a device and its serving BS.

Theoretically, if there is a perfect synchronization of time and frequency, there will be no interference and the sub-carriers will be considered orthogonal. However, in real networks, although frequency synchronization can be performed with certain accuracy, small frequency synchronization errors can still cause significant interference among different users. $f_{j,k}$ is the orthogonality factor between device *j* and device *k*.

- If device *i* and *j* decide to act as relays while device *k* transmits through D2D link, and by considering device *i* the strongest ($d_{i}\leq d_{j}$), then:
  (10) $$\left\{\begin{array}{c} Thp_{i}^{c,d}=x_{i}\rho \left(1-P_{i}^{out,cd}\left(\gamma_{i}\right)\right),\\ P_{i}^{out,cd}=1-\frac{\lambda_{j}\;e^{-\frac{\gamma_{th}\sigma_{N}^{2}\lambda_{i}}{P_{i}d_{i}^{-\alpha }}}}{\left(\lambda_{j}+\frac{\gamma_{th}P_{j}d_{j}^{-\alpha }}{P_{i}d_{i}^{-\alpha }}\lambda_{i}\right)} \end{array}\right.\left\{\begin{array}{c} Thp_{j}^{c,d}=x_{j}\rho \left(1-P_{j}^{out,cd}\left(\gamma_{j}\right)\right),\\ P_{j}^{out,cd}=1-e^{-\frac{\gamma_{th}\sigma_{N}^{2}\lambda_{j}}{P_{j}d_{j}^{-\alpha }}} \end{array}\right.$$
  (11) $$\left\{\begin{array}{c} \begin{array}{cc} Thp_{k}^{d}= & \rho \left(\left(1-x_{i}\right)\left(1-P_{i}^{out,cd}\left(\gamma_{i}\right)\right)+\left(1-x_{j}\right)\left(1-P_{j}^{out,cd}\left(\gamma_{j}\right)\right)\right)\\ & (1-P_{k}^{out,d}\left(\gamma_{k}\right)), \end{array}\\ P_{k}^{out,d}=1-e^{-\frac{\gamma_{th}\sigma_{N}^{2}\lambda_{k}}{P_{k,d}d_{k,d}^{-\alpha_{d}}}} \end{array}\right.$$
- If all devices decide to switch to D2D communication, each device earns a regret of being disconnected from the network and the throughput is given by:
  (12) $$Thp_{i}^{d}=-r_{i}$$

### 4.3. Biform Game Analysis

**Figure 3:**
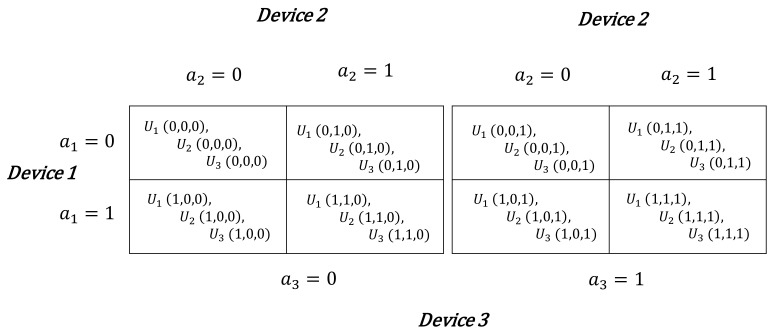
Strategic Form of the game, representing the payoffs of each device according to their choices.

Consider a two-stage decision problem of three devices. Each player *i* ’s profit (with i={1,2,3}) is its throughput as presented in Figure 3. Recall that at each transmission, each device has the choice of staying connected to the BS or instead switch to a D2D communication. A device has the right to switch to the D2D side and go back to the cellular side whenever it wants, it is a random and reversible process.

The players decide to cooperate and choose whether to be connected to the cellular or D2D link to improve their throughput. If a device stays connected to the cellular link and there is at least one device in the D2D side, the cellular device must serve as a relay to D2D devices.

**Figure 4:**
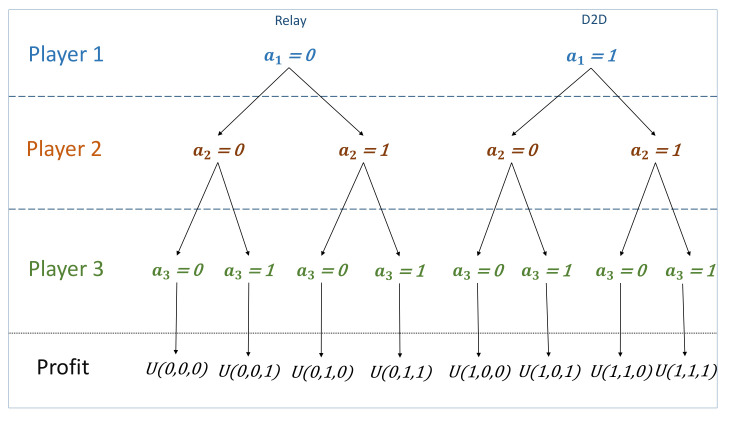
Game combination possibilities depending on each device choices.

There are $2^{3}$ different cooperation combinations between the three devices as shown in Figure 4. Depending on the devices combinations, they earn different throughput as follows:

- If all the devices decide to stay connected to cellular link, each of them earns $Thp_{i}^{c}$ as throughput.
- If at least one player switches to D2D mode, it earns $Thp_{j}^{d}$, while those who stay connected to the BS earn $Thp_{i}^{c,d}$, i≠j.
- If all the devices decide to switch to D2D communication, each of them will have $-r_{i}$ that represents regret of being disconnected from the network.

As mentioned before, biform game consists of two stages:

**First Stage**: This stage is considered as a non-cooperative game. The decision of player *i*∈{1,2,3}, is either to communicate through the cellular link and serve as relay or to communicate through D2D. This could be represented by a binary decision variable $a_{i}\in \left\{0,1\right\}$ with:

- $a_{i}=0$ refers to the choice of the action of being a relay.
- $a_{i}=1$ refers to the action of communicating through D2D.

**Second Stage**: This stage is considered as a cooperative game, where the value created U(**a**) (i.e., the characteristic function) is investigated, with **a** = $\left(a_{1},a_{2},a_{3}\right)$ refers to the decisions taken by the devices in the first stage. In other words, U(**a**) is the value (i.e., throughput profit) that the players gain as a result of cooperating in the second-stage game given that strategies $\left(a_{1},a_{2},a_{3}\right)$ were played in the first stage. To analyze the game, we start by analyzing the cooperative part and then work back to find the optimal strategy for the devices. Each case of the second-stage cooperative games has a single point core:

- The core of the game **a** = (0,0,0) is an allocation in which each player *i* gets $Thp_{i}^{c}$.
- The core of **a** = (1,0,0) is an allocation in which player 1 gets $Thp_{1}^{d}$ while player 2 and 3 get $Thp_{2}^{c,d}$ and $Thp_{3}^{c,d}$, respectively. Similarly for **a** = (0,1,0) and **a** = (0,0,1).
- The core of **a** = (1,1,0) is an allocation in which player 1 and 2 earn $Thp_{1}^{d}$ and $Thp_{2}^{d}$, respectively, while player 3 earns $Thp_{3}^{c,d}$. Similarly for **a** = (0,1,1) and **a** = (1,0,1).
- The core of the game **a** = (1,1,1) is an allocation in which each player *i* earns a regret because all the devices are disconnected totally from the BS.

Hence the second-stage in each game is deterministic as a result of first-stage devices’ decisions, as shown in Figure 4.

Note that these choices are made simultaneously. The profit that represents each device’s throughput depending on their choices is expressed as follows:

(13) $$\left\{\begin{array}{c} U\left(0,0,0\right)=\left\{Thp_{1}^{c},Thp_{2}^{c},Thp_{3}^{c}\right\}\\ U\left(0,0,1\right)=\left\{Thp_{1}^{c,d},Thp_{2}^{c,d},Thp_{3}^{d}\right\}\\ U\left(0,1,0\right)=\left\{Thp_{1}^{c,d},Thp_{2}^{d},Thp_{3}^{c,d}\right\}\\ U\left(0,1,1\right)=\left\{Thp_{1}^{c,d},Thp_{2}^{d},Thp_{3}^{d}\right\} \end{array}\right.\left\{\begin{array}{c} U\left(1,0,0\right)=\left\{Thp_{1}^{d},Thp_{2}^{c,d},Thp_{3}^{c,d}\right\}\\ U\left(1,0,1\right)=\left\{Thp_{1}^{d},Thp_{2}^{c,d},Thp_{3}^{d}\right\}\\ U\left(1,1,0\right)=\left\{Thp_{1}^{d},Thp_{2}^{d},Thp_{3}^{c,d}\right\}\\ U\left(1,1,1\right)=\left\{Thp_{1}^{d},Thp_{2}^{d},Thp_{3}^{d}\right\} \end{array}\right.$$

As explained before, there are two cases of analyzing the problem:

#### 4.3.1. First Case

The first-stage decision of player *i* is represented by a binary decision variable $a_{i}\in \left\{0,1\right\}$. In the second stage, after the first stage switching choice **a** has taken place, the corresponding cooperative game is then played. Let $U_{i}$(**a**) denotes the second stage profits for a player *i* given first stage choice **a**. The programming problem of player *i* can be written as:

(14) $$\max_{a_{i}\in \left\{0,1\right\}}E\left[U_{i}\left(a\right)\right]$$

Here the player *i* chooses the action $a_{i}$ that maximizes its second stage profit, with:

(15) $$U_{i}\left(a\right)=\left\{\begin{array}{c} Thp_{i}^{c}\;\;if\;a_{1}=a_{2}=a_{3}=0\\ Thp_{i}^{c,d}\;if\;a_{i}=0\;and\;a_{1}+a_{2}+a_{3}\leq 2\\ Thp_{i}^{d}\;if\;a_{i}=1\;and\;a_{1}+a_{2}+a_{3}\leq 2\\ -r_{i}\;if\;a_{1}=a_{2}=a_{3}=1 \end{array}\right.$$

Let us take for example the case of the player 1. Let $a_{1}$ be the binary decision variable of player 1, with $a_{1}\in \left\{0,1\right\}$. Let $\epsilon_{2},\epsilon_{3}$ be random variables representing player 2 and player 3 decision values, where $\epsilon_{2}$ and $\epsilon_{3}\in \left\{0,1\right\}$. Let $U_{1}\left(a_{1},\epsilon_{2},\epsilon_{3}\right)$ represents the second stage gain achievable by player 1 given its first stage choice $a_{1}$, player 2’s and player 3’s decisions $\epsilon_{2},\epsilon_{3}$, respectively.

The problem of player 1 can be written as:

(16) $$\max_{a_{1}\in \left\{0,1\right\}}E_{\epsilon_{2},\epsilon_{3}}\left[U_{1}\left(a_{1},\epsilon_{2},\epsilon_{3}\right)\right]$$

Note that $E_{\epsilon_{2},\epsilon_{3}}\left[U_{1}\left(a_{1},\epsilon_{2},\epsilon_{3}\right)\right]$ is the expected utility of player 1 depending on player 2 and player 3 decisions.

In Equation (16), player 1 is selecting $a_{1}$, which maximizes its second-stage expected profit. Suppose that player 1 believes that players 2 and 3 will choose to communicate through the cellular link with a probability of belief $y_{2}\geq 0$ and $y_{3}\geq 0$, respectively. So we can rewrite the above problem as:

(17) $$\max_{a_{1}\in \left\{0,1\right\}}y_{2}y_{3}\left(U_{1}\left(a_{1},1,1\right)\right)+y_{2}\left(1-y_{3}\right)\left(U_{1}\left(a_{1},1,0\right)\right)+\left(1-y_{2}\right)y_{3}\left(U_{1}\left(a_{1},0,1\right)\right)+\left(1-y_{2}\right)\left(1-y_{3}\right)\left(U_{1}\left(a_{1},0,0\right)\right).$$

In Equation (17), device 1 chooses the action that allows it to attain its maximum throughput depending on some probability beliefs it has on which action other devices can choose. The second stage throughput profit of player 1 can be written as:

(18) $$U_{1}\left(a_{1},\epsilon_{2},\epsilon_{3}\right)=\left\{\begin{array}{c} Thp_{1}^{c}\;if\;a_{1}=0\;\mathrm{and}\;\epsilon_{2}=\epsilon_{3}=0\\ Thp_{1}^{c,d}\;if\;a_{1}=0\;\mathrm{and}\;\epsilon_{2}+\epsilon_{3}\geq 1\\ Thp_{1}^{d}\;if\;a_{1}=1\;\mathrm{and}\;\epsilon_{2}+\epsilon_{3}\leq 1\\ -r_{1}\;if\;a_{1}=1\;\mathrm{and}\;\epsilon_{2}=\epsilon_{3}=1 \end{array}\right.$$

The result is that player 1 should switch to D2D if he believes that his profit in D2D is higher than his profit in cellular and vice-versa, while he is indifferent between the two options when the benefits are equal.

#### 4.3.2. Second Case

In this case, we aim to find both the pure and mixed strategy Nash equilibria that allow the devices to attain their equilibrium in terms of the highest throughput. In game theory, if each player has chosen an action strategy, and no player can benefit by modifying its strategy while the other players keep theirs unchanged, then the current set of strategy choices and their corresponding payoffs form a Nash equilibrium. Likewise, there exists a Nash equilibrium for every finite game. The Nash equilibrium could be either a pure strategy or a mixed strategy.

**Pure strategy Nash Equilibrium (PNE):** A pure strategy determines the action a device will choose with probability 1 and every other action with probability 0 to attain its best profit.

**Lemma** **1.**

- *The action (0,0,0) is a PNE iff:*
*$\left(1-P_{1}^{out,c}\left(0,0,0\right)\right)\geq \left(1-P_{1}^{out,d}\left(1,0,0\right)\right)\left(\left(1-x_{2}\right)\left(1-P_{2}^{out,cd}\left(1,0,0\right)\right)+\left(1-x_{3}\right)\left(1-P_{3}^{out,cd}\left(1,0,0\right)\right)\right)$,*
*and*
*$\left(1-P_{2}^{out,c}\left(0,0,0\right)\right)\geq \left(1-P_{2}^{out,d}\left(0,1,0\right)\right)\left(\left(1-x_{1}\right)\left(1-P_{1}^{out,cd}\left(0,1,0\right)\right)+\left(1-x_{3}\right)\left(1-P_{3}^{out,cd}\left(0,1,0\right)\right)\right)$,*
*and*
*$\left(1-P_{3}^{out,c}\left(0,0,0\right)\right)\geq \left(1-P_{3}^{out,d}\left(0,0,1\right)\right)\left(\left(1-x_{1}\right)\left(1-P_{1}^{out,cd}\left(0,0,1\right)\right)+\left(1-x_{2}\right)\left(1-P_{2}^{out,cd}\left(0,0,1\right)\right)\right)$.*
- *The action (0,0,1) is a PNE iff:*
*$x_{1}\left(1-P_{1}^{out,cd}\left(0,0,1\right)\right)\geq \frac{1-x_{2}}{2}\left(1-P_{2}^{out,cd}\left(1,0,1\right)\right)\left(1-P_{1}^{out,d}\left(1,0,1\right)\right)$,*
*and*
*$x_{2}\left(1-P_{2}^{out,cd}\left(0,0,1\right)\right)\geq \frac{1-x_{1}}{2}\left(1-P_{1}^{out,cd}\left(0,1,1\right)\right)\left(1-P_{2}^{out,d}\left(0,1,1\right)\right)$,*
*and*
*$\left(1-P_{3}^{out,d}\left(0,0,1\right)\right)\left(\left(1-x_{1}\right)\left(1-P_{1}^{out,cd}\left(0,0,1\right)\right)+\left(1-x_{2}\right)\left(1-P_{2}^{out,cd}\left(0,0,1\right)\right)\right)\geq \left(1-P_{3}^{out,c}\left(0,0,0\right)\right)$.*
- *The action (0,1,0) is a PNE iff:*
*$x_{1}\left(1-P_{1}^{out,cd}\left(0,1,0\right)\right)\geq \frac{1-x_{3}}{2}\left(1-P_{3}^{out,cd}\left(1,1,0\right)\right)\left(1-P_{1}^{out,d}\left(1,1,0\right)\right)$,*
*and*
*$\left(1-P_{2}^{out,d}\left(0,1,0\right)\right)\left(\left(1-x_{1}\right)\left(1-P_{1}^{out,cd}\left(0,1,0\right)\right)+\left(1-x_{3}\right)\left(1-P_{3}^{out,cd}\left(0,1,0\right)\right)\right)\geq \left(1-P_{2}^{out,c}\left(0,0,0\right)\right)$,*
*and*
*$x_{3}\left(1-P_{3}^{out,cd}\left(0,1,0\right)\right)\geq \frac{1-x_{1}}{2}\left(1-P_{1}^{out,cd}\left(0,1,1\right)\right)\left(1-P_{3}^{out,d}\left(0,1,1\right)\right)$.*
- *The action (0,1,1) is a PNE iff:*
*$x_{1}\rho \left(1-P_{1}^{out,cd}\left(0,1,1\right)\right)\geq -r_{1}$,*
*and*
*$\frac{1-x_{1}}{2}\left(1-P_{1}^{out,cd}\left(0,1,1\right)\right)\left(1-P_{2}^{out,d}\left(0,1,1\right)\right)\geq x_{2}\left(1-P_{2}^{out,cd}\left(0,0,1\right)\right)$,*
*and*
*$\frac{1-x_{1}}{2}\left(1-P_{1}^{out,cd}\left(0,1,1\right)\right)\left(1-P_{3}^{out,d}\left(0,1,1\right)\right)\geq x_{3}\left(1-P_{3}^{out,cd}\left(0,1,0\right)\right)$.*
- *The action (1,0,0) is a PNE iff:*
*$\left(1-P_{1}^{out,d}\left(1,0,0\right)\right)\left(\left(1-x_{2}\right)\left(1-P_{2}^{out,cd}\left(1,0,0\right)\right)+\left(1-x_{3}\right)\left(1-P_{3}^{out,cd}\left(1,0,0\right)\right)\right)\geq \left(1-P_{1}^{out,c}\left(0,0,0\right)\right)$,*
*and*
*$x_{2}\left(1-P_{2}^{out,cd}\left(1,0,0\right)\right)\geq \frac{1-x_{3}}{2}\left(1-P_{3}^{out,cd}\left(1,1,0\right)\right)\left(1-P_{2}^{out,d}\left(1,0,0\right)\right)$,*
*and*
*$x_{3}\left(1-P_{3}^{out,cd}\left(1,0,0\right)\right)\geq \frac{1-x_{2}}{2}\left(1-P_{2}^{out,cd}\left(1,0,1\right)\right)\left(1-P_{3}^{out,d}\left(1,0,1\right)\right)$,*
- *The action (1,1,0) is a PNE iff:*
*$\frac{1-x_{3}}{2}\left(1-P_{3}^{out,cd}\left(1,1,0\right)\right)\left(1-P_{1}^{out,d}\left(1,1,0\right)\right)\geq x_{1}\left(1-P_{1}^{out,cd}\left(0,1,0\right)\right)$,*
*and*
*$\frac{1-x_{3}}{2}\left(1-P_{3}^{out,cd}\left(1,1,0\right)\right)\left(1-P_{2}^{out,d}\left(1,1,0\right)\right)\geq x_{2}\left(1-P_{2}^{out,cd}\left(1,0,0\right)\right)$,*
*and*
*$x_{3}\left(1-P_{3}^{out,cd}\left(1,1,0\right)\right)\geq -r_{3}$.*
- *The action (1,0,1) is a PNE iff:*
*$\frac{1-x_{2}}{2}\left(1-P_{2}^{out,cd}\left(1,0,1\right)\right)\left(1-P_{2}^{out,d}\left(1,0,1\right)\right)\geq x_{1}\left(1-P_{1}^{out,cd}\left(0,0,1\right)\right)$,*
*and*
*$x_{2}\left(1-P_{2}^{out,cd}\left(1,0,1\right)\right)\geq -r_{2}$,*
*and*
*$\frac{1-x_{2}}{2}\left(1-P_{2}^{out,cd}\left(1,0,1\right)\right)\left(1-P_{3}^{out,d}\left(1,0,1\right)\right)\geq x_{3}\left(1-P_{3}^{out,cd}\left(1,0,0\right)\right)$.*
- *The action (1,1,1) is a PNE iff:*
*$-r_{1}\geq x_{1}\left(1-P_{1}^{out,cd}\left(0,1,1\right)\right)$,*
*and*
*$-r_{2}\geq x_{2}\left(1-P_{2}^{out,cd}\left(1,0,1\right)\right)$,*
*and*
*$-r_{3}\geq x_{3}\left(1-P_{3}^{out,cd}\left(1,1,0\right)\right)$.*
*One can clearly see that the action strategy (1,1,1) could never be a PNE. This is because the throughput could not be a negative value.*

**Proof.**  See Appendix A.1 □

Different from the pure equilibria analysis, where we consider unknown, slow fading and stationary channels, in the mixed analysis we consider random and fast fading channels. In a fast fading channel, a device can find itself unable to reach a pure equilibrium strategy in some situations, but it can attain the equilibrium by adopting each strategy with a certain probability.

**Mixed strategy Nash Equilibrium (MNE):** A mixed strategy is an attribution of a probability to each pure strategy, i.e., a device chooses an action with a certain probability. A pure strategy can be considered as a degenerate case of a mixed strategy. Let $p_{i}$ denotes the probability of relaying of each device *i*, so $\left(1-p_{i}\right)$ is its probability of choosing to communicate through D2D.

- If player 1 is indifferent between choosing to be a relay or to switch to D2D, then:
  $E\left[U_{1}\left(0,a_{2},a_{3}\right)\right]=E\left[U_{1}\left(1,a_{2},a_{3}\right)\right]$, with:

(19) $$\left\{\begin{array}{c} E\left[U_{1}\left(0,a_{2},a_{3}\right)\right]=U_{1}\left(0,0,0\right)p_{2}p_{3}+U_{1}\left(0,1,0\right)\left(1-p_{2}\right)p_{3}+U_{1}\left(0,0,1\right)p_{2}\left(1-p_{3}\right)+U_{1}\left(0,1,1\right)\left(1-p_{2}\right)\left(1-p_{3}\right)\\ E\left[U_{1}\left(1,a_{2},a_{3}\right)\right]=U_{1}\left(1,0,0\right)p_{2}p_{3}+U_{1}\left(1,1,0\right)\left(1-p_{2}\right)p_{3}+U_{1}\left(1,0,1\right)p_{2}\left(1-p_{3}\right)+U_{1}\left(1,1,1\right)\left(1-p_{2}\right)\left(1-p_{3}\right) \end{array}\right.$$

- If player 2 is indifferent between choosing to be a relay or to switch to D2D, then:
  $E\left[U_{2}\left(a_{1},0,a_{3}\right)\right]=E\left[U_{2}\left(a_{1},1,a_{3}\right)\right]$, with:

(20) $$\left\{\begin{array}{c} E\left[U_{2}\left(a_{1},0,a_{3}\right)\right]=U_{2}\left(0,0,0\right)p_{1}p_{3}+U_{2}\left(1,0,0\right)\left(1-p_{1}\right)p_{3}+U_{2}\left(0,0,1\right)p_{1}\left(1-p_{3}\right)+U_{2}\left(1,0,1\right)\left(1-p_{1}\right)\left(1-p_{3}\right)\\ E\left[U_{2}\left(a_{1},1,a_{3}\right)\right]=U_{2}\left(0,1,0\right)p_{1}p_{3}+U_{2}\left(1,1,0\right)\left(1-p_{1}\right)p_{3}+U_{2}\left(0,1,1\right)p_{1}\left(1-p_{3}\right)+U_{2}\left(1,1,1\right)\left(1-p_{1}\right)\left(1-p_{3}\right) \end{array}\right.$$

- If player 3 is indifferent between choosing to be a relay or to switch to D2D, then:
  $E\left[U_{3}\left(a_{1},a_{2},0\right)\right]=E\left[U_{3}\left(a_{1},a_{2},1\right)\right]$, with:

(21) $$\left\{\begin{array}{c} E\left[U_{3}\left(a_{1},a_{2},0\right)\right]=U_{3}\left(0,0,0\right)p_{1}p_{2}+U_{3}\left(1,0,0\right)\left(1-p_{1}\right)p_{2}+U_{3}\left(0,1,0\right)p_{1}\left(1-p_{2}\right)+U_{3}\left(1,1,0\right)\left(1-p_{1}\right)\left(1-p_{2}\right)\\ E\left[U_{3}\left(a_{1},a_{2},1\right)\right]=U_{3}\left(0,0,1\right)p_{1}p_{2}+U_{3}\left(1,0,1\right)\left(1-p_{1}\right)p_{2}+U_{3}\left(0,1,1\right)p_{1}\left(1-p_{2}\right)+U_{3}\left(1,1,1\right)\left(1-p_{1}\right)\left(1-p_{2}\right) \end{array}\right.$$

Then, the equilibrium probability vector $p^{*}=\left(p_{1}^{*},p_{2}^{*},p_{3}^{*}\right)$ could be obtained by solving the following system of equations:

(22) $$\left\{\begin{array}{c} E\left[U_{1}\left(0,a_{2},a_{3}\right)\right]=E\left[U_{1}\left(1,a_{2},a_{3}\right)\right],\\ E\left[U_{2}\left(a_{1},0,a_{3}\right)\right]=E\left[U_{2}\left(a_{1},1,a_{3}\right)\right],\\ E\left[U_{3}\left(a_{1},a_{2},0\right)\right]=E\left[U_{3}\left(a_{1},a_{2},1\right)\right]. \end{array}\right.$$

## 5. Equilibrium Analysis for n-Person Game

Consider a two-stage decision problem of a fixed number *n* of devices inside a single cell. At each step of the game, each of the players chooses an action. The result of each play is a random payoff defined as the throughput of each player i∈N. Depending on the devices choices of belonging to cellular or D2D group, each device earns a throughput as follows:

- If all the devices are in cellular, then the throughput of each device is:
  (23) $$\left\{\begin{array}{c} Thp_{i}^{c}=\Theta_{i}^{c}\left(\gamma_{i}\right)=\rho \left(1-P_{i}^{out,c}\left(\gamma_{i}\right)\right),\\ P_{i}^{out,c}=1-\frac{\left(\prod_{j=i+1}^{n_{c}}\lambda_{j}\right)e^{-\frac{\gamma_{i,th}\sigma_{N}^{2}\lambda_{i}}{P_{i}d_{i}^{-\alpha }}}}{\prod_{j=i+1}^{n_{c}}\left(\lambda_{j}+\frac{\gamma_{i,th}P_{j}d_{j}^{-\alpha }}{P_{i}d_{i}^{-\alpha }}\lambda_{i}\right)}. \end{array}\right.$$
- If there are $N_{c}=\left\{1,2,...,n_{c}\right\}$ devices in cellular and $N_{d}=\left\{1,2,...,n_{d}\right\}$ devices in D2D, then each device *i* in cellular has:
  (24) $$\left\{\begin{array}{c} Thp_{i}^{c,d}=x_{i}\Theta_{i}^{c,d}\left(\gamma_{i}\right)=x_{i}\rho \left(1-P_{i}^{out,cd}\left(\gamma_{i}\right)\right),\\ P_{i}^{out,cd}=1-\frac{\left(\prod_{j=i+1}^{n_{c}}\lambda_{j}\right)e^{-\frac{\gamma_{i,th}\sigma_{N}^{2}\lambda_{i}}{P_{i}d_{i}^{-\alpha }}}}{\prod_{j=i+1}^{n_{c}}\left(\lambda_{j}+\frac{\gamma_{i,th}P_{j}d_{j}^{-\alpha }}{P_{i}d_{i}^{-\alpha }}\lambda_{i}\right)} \end{array}\right.$$
  On the other hand, each device *k* in D2D group communicates with the following throughput:
  (25) $$\left\{\begin{array}{c} Thp_{k}^{d}=\frac{\sum_{i=1}^{n_{c}}\left(1-x_{i}\right)\rho \left(1-P_{i}^{out,cd}\left(\gamma_{i}\right)\right)}{N_{d}}\left(1-P_{k}^{out,d}\left(\gamma_{k}\right)\right),\\ P_{k}^{out,d}=1-\frac{\left(\prod_{j\in N_{d}\backslash \left\{k\right\}}\lambda_{j}\right)\;e^{-\frac{\gamma_{th}\sigma_{N}^{2}\lambda_{k}}{P_{k,d}d_{k,d}^{-\alpha }}}}{\prod_{j\in N_{d}\backslash \left\{k\right\}}\left(\lambda_{k}+\frac{\gamma_{th}f_{k,j}P_{j,d}d_{j,d}^{-\alpha }}{P_{k,d}d_{k,d}^{-\alpha_{d}}}\lambda_{j}\right)} \end{array}\right.$$
  We assume that the fraction of throughput given from the cellular devices is equally divided between devices in D2D.
- If all devices decide to switch to D2D communication, each device earns a regret, because there is no link left with the BS so all transmissions fail:
  (26) $$Thp_{i}^{d}=-r_{i}$$

At each transmission, each device has the choice of staying connected to the BS and serves as a relay or instead switch to D2D communication. A device has the right to join either the cellular or the D2D group whenever it wants to maximize its profit. Once in the cellular group, all the devices serve as relays to the D2D-transmitters in the other group. There are $2^{n}$ different cooperation combinations between the *n* devices inside the cell. Either all of them are communicating through cellular links, or all the devices choose to join the D2D group, or some devices communicate through cellular and serve as relays to others in the D2D group.

In the first stage, the decision of player *i* ∈N, is to choose the mode of communication. In the second stage, we investigate the throughput U(**a**) that players generate as a result of cooperating in the second-stage game given that strategies **a** = $\left(a_{1},a_{2},...,a_{n}\right)$ were played in the first stage. Then, let us denote U(**a**) as a second stage cooperative game. For example, U(0,0,...,0) is the case where all devices are in the cellular group while U(1,1,...,1) is the case where all devices choose to join the D2D group.

### 5.1. First Case

For each device *i*, $a_{i}$ is its binary decision variable, with $a_{i}\in \left\{0,1\right\}$. Let $\epsilon_{k}\in \left\{0,1\right\}$ be the decision of device k∈{1,...,n}\{i}. $U_{i}\left(a_{i},\epsilon_{k}\right)$ denotes the second stage profit achievable by device *i* given its first stage action and other devices decisions.

The problem of device *i* can be written as follows:

(27) $$\max_{a_{i}\in \left\{0,1\right\}}E_{\epsilon_{k_{\left(k\in \{1,...,n\}\backslash \{i\}\right)}}}\left[U_{i}\left(a_{i},\epsilon_{k}\right)\right],$$

where $E_{\epsilon_{k_{\left(k\in \{1,...,n\}\backslash \{i\}\right)}}}\left[U_{i}\left(a_{i},\epsilon_{k}\right)\right]$ is the expected value of device *i* when choosing action $a_{i}$ depending on the other devices decisions.

Here the player *i* chooses the action $a_{i}$ that maximizes its second stage earning, with:

(28) $$U_{i}\left(a_{i},\epsilon_{k}\right)=\left\{\begin{array}{c} Thp_{i}^{c}\;\;if\;a_{i}=0\;and\;\epsilon_{k}=0\\ Thp_{i}^{c,d}\;if\;a_{i}=0\;and\;\sum \epsilon_{k}\geq 1\\ Thp_{i}^{d}\;if\;a_{i}=1\;and\;\sum \epsilon_{k}\leq n-2\\ -r_{i}\;\;if\;a_{i}=1\;and\;\epsilon_{k}=1 \end{array}\right.$$

### 5.2. Second Case

In this case, we aim to find the PNE and the MNE of the *n*-device game. The concept of NE is used to describe a strategy as the most rational behavior by players acting to maximize their gains.

**Definition** **1.** *The strategy profile* $A^{*}$ =$\left(a_{i}^{*},a_{-i}^{*}\right)$ is a pure Nash equilibrium if and only if:

(29) $$\forall \;i\in N,\forall \;a_{i}\in A_{i}\;U_{i}\left(A^{*}\right)\geq U_{i}\left(a_{i},A_{-i}^{*}\right).$$

Nonetheless, a finite game might not always have a PNE, but it always has a MNE.

**Definition** **2.** A mixed action profile $p^{*}\in ]0,1[$ is a mixed Nash equilibrium if for each player i∈{1,2,...,n},

(30) $$p_{i}^{*}\in_{p_{i}\in \Delta \left(A_{i}\right)}\;U_{i}\left(p_{i},p_{-i}^{*}\right),$$

*where $p_{i}$ is a mixed action for player i and $p_{-i}$ is the profile of mixed actions for all players other than i. $\Delta (A_{i})$ is the set of all probability distributions over $A_{i}$, which is the set of player i pure strategies.*

From the network designer, the solutions produced by the biform game framework require complete network information, which may not scale well with the network size, and might cause high overload. Thus, for networks with incomplete information, the devices need to be self-organized and use decentralized learning algorithms to reach their equilibrium strategies. This only requires a minimal signaling to the users, and no recommendation from the BS. Part II [12] of this work covers the distributed schemes enabling the devices to reach Nash equilibrium, only based on their local information and observations.

## 6. Performance Analysis

**Figure 5:**
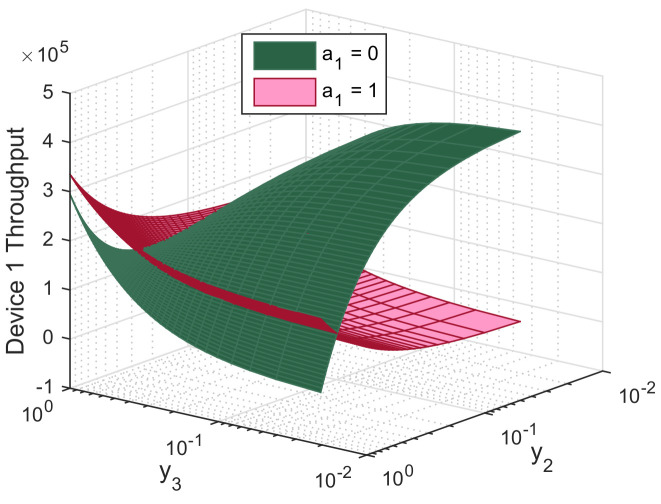
Throughput of device 1 as function of its beliefs on the relaying probabilities of device 2 ($y_{2}$) and device 3 ($y_{3}$), both when relaying ($a_{1}=0$) and not relaying ($a_{1}=1$).

**Figure 6:**
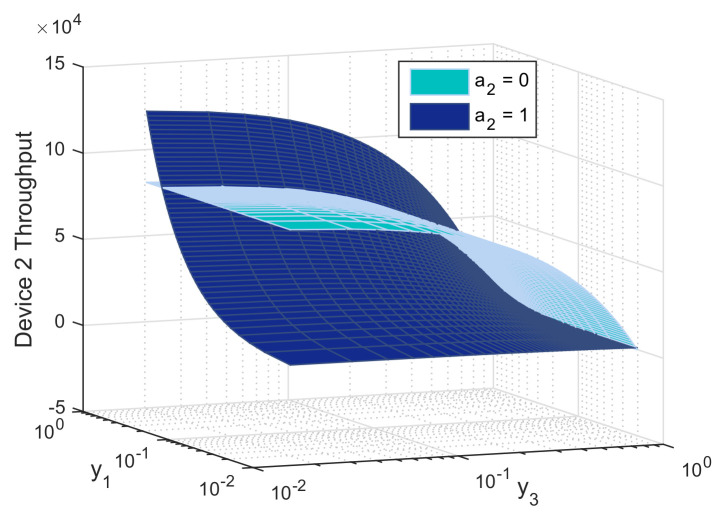
Throughput of device 2 as function of its beliefs on the relaying probabilities of device 1 ($y_{1}$) and device 3 ($y_{3}$), both when relaying ($a_{2}=0$) and not relaying ($a_{2}=1$).

**Figure 7:**
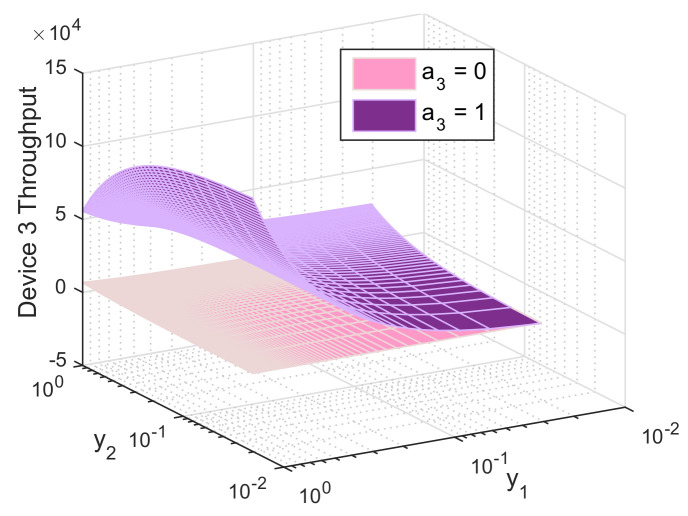
Throughput of device 3 as function of its beliefs on the relaying probabilities of device 1 ($y_{1}$) and device 3 ($y_{3}$), both when relaying ($a_{3}=0$) and not relaying ($a_{3}=1$).

In this section, we evaluate the performance of the biform game using Mathworks Matlab R2020a. For illustrative purpose, we perform simulations for the three-device case. Figures are produced using the following setup: $P_{1}^{c}=$ 10 mW, $P_{2}^{c}=$ 30 mW, $P_{3}^{c}=$ 50 mW, $P^{d}=$ 5 mW, *R* = 1 Mbit/s, *L* = *M* = 1024 bits, $\gamma_{th}$ = 40 dB, $\alpha_{c}=\alpha_{d}=3$ and $\sigma_{N}^{2}=-116$ dBm, $d_{1}=100$ m, $d_{2}=300$ m, $d_{3}=500$ m, $f=10^{-5}$, $x_{1}=x_{2}=x_{3}=0.5$, ${\left|h_{1}\right|}^{2}=0.6$, ${\left|h_{2}\right|}^{2}=0.5$, and ${\left|h_{3}\right|}^{2}=0.2$. Figure 5, Figure 6 and Figure 7 report the action that a device might choose to maximize its expected utility depending on its belief on its competitors.

Figure 5 shows that when the strongest device 1 believes devices 2 and 3 have a low chance to relay data, it has incentives to act as a relay to maximize its expected utility. Meanwhile, it is more likely to communicate over a D2D link when it believes its competitors are likely to serve as relays. We notice that the maximum throughput for device 1 is attained when it acts as relay while the other devices have a high chance to communicate through D2D. Here, it prevents earning regrets by being disconnected from the mobile service and it gets rid of all interference from the other two devices. Moreover, device 1 chooses to communicate through D2D to maximize its utility if it believes one of its competitors might be a relay. This way, it gets rid of cellular interference and transmits at lower power.

Figure 6 depicts the average throughput of device 2 while changing its beliefs on the other devices willingness to relay. We notice that the relaying probability of device 2 increases when the relaying probability of the weakest device 3 decreases. This can be explained as follows: device 3 may harm the second strongest device while transmitting over cellular link, while it is indifferent about device 1 strategy. It switches to D2D when the relaying probability of device 1 increases and that of device 3 decreases. Following this behavior, device 2 is able to get rid of high interference in cellular, transmit at lower power, use better RAN and experience satisfactory QoS brought by the strongest device.

Similarly, Figure 7 depicts the average throughput of the weakest device. When this latter decides to serve as a relay, it will experience low QoS due to the long distance and the bad channel gain leading to the BS. It also has to transmit with high power and share its throughput with other devices via D2D. However, switching to D2D allows it to benefit from a better channel quality, to transmit at lower power and to experience improved QoS offered from the stronger relays. We notice that device 3 might experience high throughput when the strongest device serves as a relay and device 2 uses D2D. In this case, device 1 gets rid of interference and perceives high throughput, meanwhile device 3 gets a fraction of that throughput, resulting in a win-win scenario.

## 7. Conclusions and Perspectives

In this article, we considered the uplink case of *n* devices, where each device chooses whether to communicate through cellular (e.g., 5G/6G) or via D2D link to maximize its throughput. Cellular devices use NOMA, whilst they may serve neighboring devices using an orthogonal multiple access method (e.g., OFDMA/SC-FDMA). We formulated the problem as a biform game: Step 1) the devices competed over two available radio access technologies (cellular and D2D); Step 2) Devices connected to cellular cooperate with other devices in order to provide access to available services. Next, we analyzed the game pure/mixed equilibria. Simulation results show that D2D-relaying improves the devices’ average throughput. The second part of this article [12] deals with implementing distributed reinforcement learning to self-explore optimal strategies in a fully distributed manner.

## Figures and Tables

**Table 1:** Related works on Device-to-Device (D2D) mode selection vs. our work.

Ref	D2D Mode	Multiple Access	Main Goal	Tools	UEs Access Mode
[32]	Underlay	OMA	Improve Cellular Coverage Quality	Optimization mechanism Greedy algorithm based on a distributed local search	- Cellular mode - Multi-hop D2D relaying mode
[33]	Overlay	OMA	Minimize the average energy consumption of flow transmission	Markov Decision Process	- Cellular mode - D2D mode
[34]	Underlay	OMA	Achieve high spectrum efficiency	Evolutionary game model	- Cellular mode - Direct reuse mode - D2D Relay mode
[35]	Underlay	OMA	Optimize the network energy efficiency- Maximize the number of connected D2D users	Fuzzy C mean - clustering algorithm	- Dedicated D2D mode - D2D reuse mode
[36]	Underlay	OMA	Increase the data rate Improve the energy efficiency- Satisfy stringent delay constraints	Energy Efficiency and Delay-Optimization algorithm based on the brute-force searching method	- Direct transmission - D2D-assisted relaying
[37]	Outband Underlay	Listen-before- talk (LBT)/ Duty-cycle method	Minimize the mutual interference - Guarantee the QoS requirements- Maximize the overall throughput	Heuristic algorithms	- Licensed reusing mode - Duty-cycle based - LBT based unlicensed modes
[38]	Underlay	OMA	Maximize the system throughput	Mode Selection and Resource Allocation algorithm based on Lagrangian dual decomposition	- Cellular mode - D2D mode
[39]	Underlay Overlay	OMA	Optimize the total throughput- Reduce interference	Probabilistic integrated resource allocation strategy Quasi-convex optimization algorithm	- Reusing Mode - Dedicated Mode - Cellular Mode
[40]	Underlay	OMA	Maximize the number of D2D users - Increase the system capacity - Improve the overall throughput	Greedy algorithm Heuristic algorithm	- Cellular mode - Direct D2D mode
Our work	Overlay	NOMA OMA	Pure and Mixed Equilibrium in terms of throughput and reliability	Game theory Distributed reinforcement learning	- Cellular mode - Relay mode - D2D mode

**Table 2:** Main symbols and their meanings.

Symbol	Meaning
*n*	Number of devices in the cell
$P_{i}$	Transmission power of device *i*
$d_{i}$	Distance between device *i* and the BS
$h_{i}$	Channel gain of device *i*
$\gamma_{i}$	SINR of device *i*
$\gamma_{i,th}$	SINR-threshold
$P_{i}^{out}\left(\gamma_{i}\right)$	Outage probability of device *i*
$\frac{1}{\lambda }$	Mean of the channel gain
*R*	Transmission rate
$P_{i}^{out,c}$	Outage probability of device *i* if it communicates through cellular
$P_{i}^{out,cd}$	Outage probability of device *i* if it is a relay
$P_{i}^{out,d}$	Outage probability of device *i* if it communicates through D2D
$Thp_{i}^{c}$	Throughput of device *i* if it communicates through cellular
$Thp_{i}^{c,d}$	Throughput of device *i* if it is a relay
$Thp_{i}^{d}$	Throughput of device *i* if it communicates through D2D
$P_{i,d}$	Transmission power of device *i* if it communicates through D2D
$d_{i,d}$	Distance between device *i* and another D2D device
*f*	Orthogonality factor
$\alpha_{c},\alpha_{d}$	Path-loss exponent in cellular and D2D, respectively
$x_{i}$	Fraction of throughput device *i* gives to D2D devices
$U_{i}\left(a_{i}\right)$	Utility of device *i* that denotes its throughput when choosing the action $a_{i}$

## Appendix A

### Appendix A.1. Proof of Lemma 1

The proof is straightforward by applying the definition of pure Nash equilibrium.

- The action (0,0,0) is a PNE iff:
  $U_{1}\left(0,0,0\right)\geq U_{1}\left(1,0,0\right)$ and $U_{2}\left(0,0,0\right)\geq U_{2}\left(0,1,0\right)$ and $U_{3}\left(0,0,0\right)\geq U_{3}\left(0,0,1\right)$
- The action (0,1,0) is a PNE iff:
  $U_{1}\left(0,1,0\right)\geq U_{1}\left(1,1,0\right)$ and $U_{2}\left(0,1,0\right)\geq U_{2}\left(0,0,0\right)$ and $U_{3}\left(0,1,0\right)\geq U_{3}\left(0,1,1\right)$
- The action (0,0,1) is a PNE iff:
  $U_{1}\left(0,0,1\right)\geq U_{1}\left(1,0,1\right)$ and $U_{2}\left(0,0,1\right)\geq U_{2}\left(0,1,1\right)$ and $U_{3}\left(0,0,1\right)\geq U_{3}\left(0,0,0\right)$
- The action (0,1,1) is a PNE iff:
  $U_{1}\left(0,1,1\right)\geq U_{1}\left(1,1,1\right)$ and $U_{2}\left(0,1,1\right)\geq U_{2}\left(0,0,1\right)$ and $U_{3}\left(0,1,1\right)\geq U_{3}\left(0,1,0\right)$
- The action (1,0,0) is a PNE iff:
  $U_{1}\left(1,0,0\right)\geq U_{1}\left(0,0,0\right)$ and $U_{2}\left(1,0,0\right)\geq U_{2}\left(1,1,0\right)$ and $U_{3}\left(1,0,0\right)\geq U_{3}\left(1,0,1\right)$
- The action (1,1,0) is a PNE iff:
  $U_{1}\left(1,1,0\right)\geq U_{1}\left(0,1,0\right)$ and $U_{2}\left(1,1,0\right)\geq U_{2}\left(1,0,0\right)$ and $U_{3}\left(1,1,0\right)\geq U_{3}\left(1,1,1\right)$
- The action (1,0,1) is a PNE iff:
  $U_{1}\left(1,0,1\right)\geq U_{1}\left(0,0,1\right)$ and $U_{2}\left(1,0,1\right)\geq U_{2}\left(1,1,1\right)$ and $U_{3}\left(1,0,1\right)\geq U_{3}\left(1,0,0\right)$
- The action (1,1,1) is a PNE iff:
  $U_{1}\left(1,1,1\right)\geq U_{1}\left(0,1,1\right)$ and $U_{2}\left(1,1,1\right)\geq U_{2}\left(1,0,1\right)$ and $U_{3}\left(1,1,1\right)\geq U_{3}\left(1,1,0\right)$
